# Survival Prediction in Gallbladder Cancer Using CT Based Machine Learning

**DOI:** 10.3389/fonc.2020.604288

**Published:** 2020-11-27

**Authors:** Zefan Liu, Guannan Zhu, Xian Jiang, Yunuo Zhao, Hao Zeng, Jing Jing, Xuelei Ma

**Affiliations:** ^1^Laboratory of Tumor Targeted and Immune Therapy, Clinical Research Center for Breast, West China Hospital, Sichuan University, Chengdu, China; ^2^State Key Laboratory of Biotherapy, Department of Biotherapy, Cancer Center, West China Hospital, Sichuan University, Chengdu, China

**Keywords:** radiomics, machine learning, gallbladder cancer, prognosis, random forest

## Abstract

**Objective:**

To establish a classifier for accurately predicting the overall survival of gallbladder cancer (GBC) patients by analyzing pre-treatment CT images using machine learning technology.

**Methods:**

This retrospective study included 141 patients with pathologically confirmed GBC. After obtaining the pre-treatment CT images, manual segmentation of the tumor lesion was performed and LIFEx package was used to extract the tumor signature. Next, LASSO and Random Forest methods were used to optimize and model. Finally, the clinical information was combined to accurately predict the survival outcomes of GBC patients.

**Results:**

Fifteen CT features were selected through LASSO and random forest. On the basis of relative importance GLZLM-HGZE, GLCM-homogeneity and NGLDM-coarseness were included in the final model. The hazard ratio of the CT-based model was 1.462(95% CI: 1.014–2.107). According to the median of risk score, all patients were divided into high and low risk groups, and survival analysis showed that high-risk groups had a poor survival outcome (*P* = 0.012). After inclusion of clinical factors, we used multivariate COX to classify patients with GBC. The AUC values in the test set and validation set for 3 years reached 0.79 and 0.73, respectively.

**Conclusion:**

GBC survival outcomes could be predicted by radiomics based on LASSO and Random Forest.

## Introduction

Gallbladder cancer (GBC) is the fifth most common tumor of the digestive system and accounts for 95% of malignant tumors in the biliary system (1). The lack of specific clinical manifestations in the early stage, coupled with high invasive biological features and abnormal anatomic location of the gallbladder, results in poor survival outcomes (2, 3). In addition, due to the low sensitivity to chemotherapy and radiotherapy, and the lack of effective therapeutic targets, surgical resection is still the main treatment option (4). At present, widespread concern about the survival outcomes of GBC has been aroused. Researchers hope to distinguish patients with higher prognostic risk, so as to implement personalized medical treatment. So far, prognostic analyses of GBC have depended on laboratory-tested indicators such as tumor markers, nutritional indicators, and gene expression signatures, but these indicators lack an intuitive analysis of the whole tumor lesion (5–7).

Lesions can be directly observed by radiological images in clinic and lesion information not visible to the naked eye can be provided by radiomics. In recent years, radiomics has been developed to focus on the extraction and mining massive medical imaging data. It is hypothesized that these selected imaging features reflect specific tumor phenotypes (8, 9). Because these image signatures provide a comprehensive picture of the entire tumor entity, the heterogeneity of these signatures may have implications for clinical events such as treatment response, survival outcomes and disease progression. Some studies have focused on the appearance of imaging features at different cancer stages (10, 11). In addition many other studies have reported the effect of imaging features on survival outcomes, but no studies have been reported on GBC.

GBC survival prediction model is of great significance for patients’ prognosis assessment, treatment mode selection, surgical patient selection, postoperative adjuvant treatment plan determination, high-risk recurrence patient identification, follow-up frequency formulation, and rational use of medical resources. In this study, we assessed a number of CT-based radiomics parameters to predict patient’s overall survival (OS). Patient cohort with a total of 141 patients was used to analyze image data, extract features, and perform model tests. All the selected parameters were evaluated for their predictive power and stability. Finally, we combined clinical information for a cost-effective prediction.

## Methods

### Patient Selection

**Figure 1:**
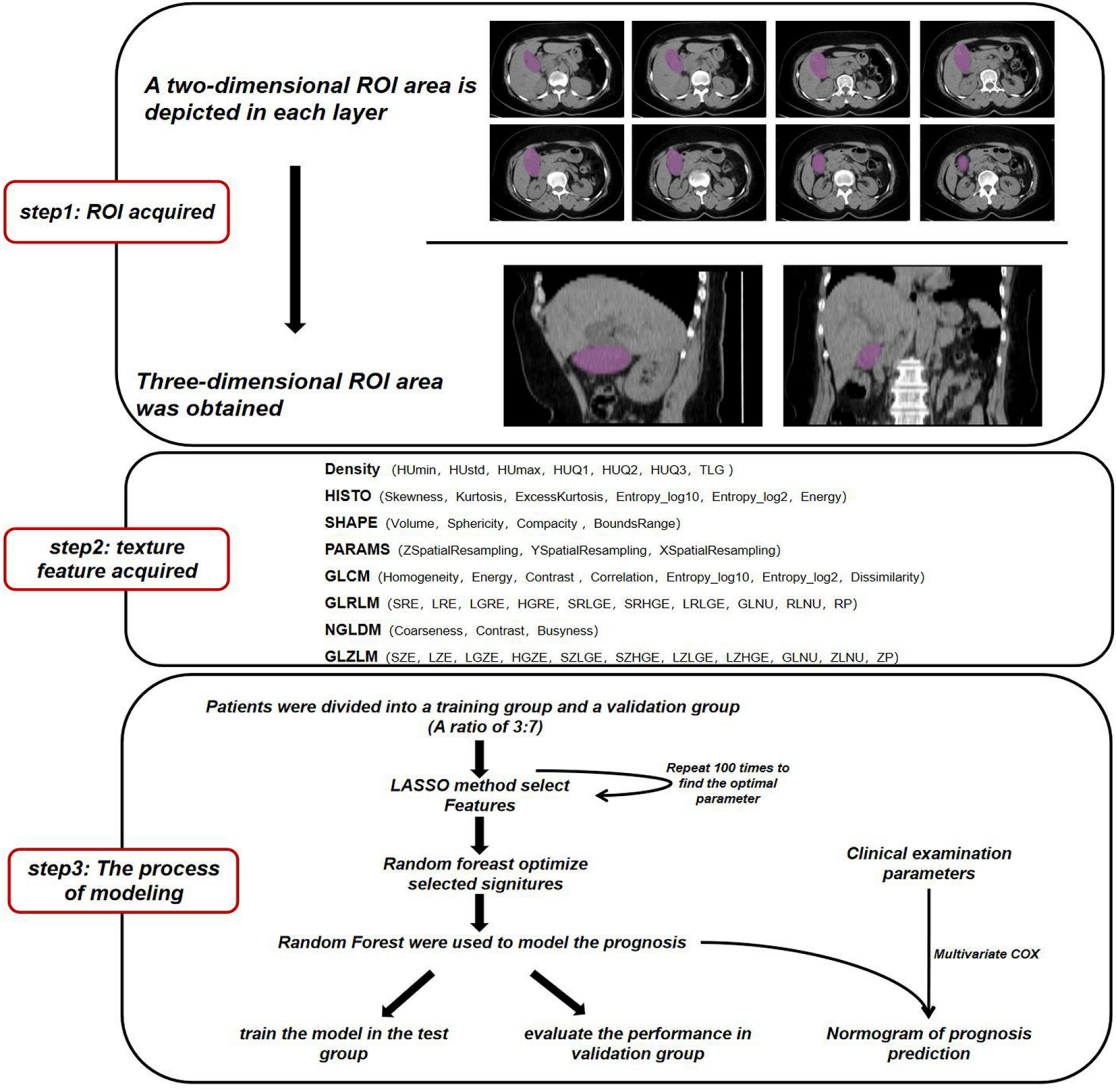
Workflow for image processing and machine learning.

The flow of data analysis and processing is shown in **Figure 1**. The records of patients from 2010 to 2017 were selected from the Department of Hepatobiliary surgery through an electronic medical review. Inclusion criteria for patients: 1) Pathological examination Confirmed GBC; 2) Perform CT scan before tumor biopsy or surgery. Some patients were excluded because of the history of liver surgery or other liver lesions leading to gallbladder lesions that could not be identified (**Table 1**). Considering that conventional CT, including CT and Contrast-enhanced CT, is commonly used tests in clinical practice and have good cost-effectiveness, it was selected as the study object. Finally, a total of 141 patients were included in this study, and CT images were collected from Radiology Department.

**Table 1:** The general condition of the patients in this study.

Patient (Total=141)	Patient characteristics		
	Male	Female	
Gender	56(39.7%)	85(60.3%)	
	<30	30-50	>50
Age	23(16.3%)	84(59.5%)	34(24.1%)
	I-II	III-IV	
T stage	49(32.8%)	92(65.2)	
	N0	N1	N2
N stage	67(47.5%)	56(39.7%)	18(12.7%)
	M0	M1	
M stage	82(58.1%)	56(39.7)	
	Yes	No	
Liver metastasis	78(55.3%)	63(44.6%)	
	Yes	No	
Jaundice	33(23.4%)	108(76.5%)	
	<40 u/ml	>=40 u/ml	NA
CA199	59(41.8%)	79(56.0%)	3(2.12%)
	<35 u/ml	>=35 u/ml	NA
CA125	85(60.2%)	53(37.5%)	3(2.12%)
	<5 μg/L	>=5 μg/L	NA
CEA	90(63.8%)	48(34.0%)	3(2.12%)
	<20 μg/L	>=20 μg/L	NA
AFP	130(92.1%)	8(5.67%)	3(2.12%)
	Yes	No	
Surgical treatment	114(80.8%)	27(19.1%)	

All procedures involving human participants comply with ethical standards bodies and/or national research councils. Ethics Committee of Sichuan University approved this retrospective study. Written informed consent (written informed consent for patients under 16 years of age must be signed by a parent or guardian) is required before radiological examination for all patients.

### Image Recognition and Feature Extraction

CT scanning was performed using 64-MDCT Scanner (Brilliance64, Philips Medical Systems, Eindhoven, The Netherlands) or 128-MDCT scanner (Somatom Definition AS+, Siemens Healthcare Sector, Forchheim, Germany) before going through any treatment.

All CT examinations were performed under the following conditions: 120 kVp; 199 mAs; 12.9 ctdIVOL (mGy); 460.7 DLP (mGy*cm); pitch, 0.75–1.0; rotation time, 0.5–0.75 s; collimation, 0.625 mm; section thickness, 2.0 or 5.0 mm.

The ROI area was sketched by two experienced radiologists.Due to the limited recognition ability of ordinary CT for GBC and the boundary of cancer is usually fuzzy, we followed the following principle when making segmentation: 1) delineate solid lesions with high density of GBC and avoid low-density areas such as bile, 2) delineate the definite tumor part when it is difficult to recognize the blurring around the lesion, and 3) excluded samples with disagreement among radiologists.

We used the image feature extraction software LIFEx to obtain the texture signatures of CT images (12). Based on each layer of CT image, we depicted the boundary of the lesion in the two-dimensional region of interest (ROI) and finally obtained a three-dimensional ROI. ROI is described by independent radiologists who do not know the patient’s diagnosis (**Supplement Figure 1**). The maximum, minimum, mean, and standard deviation of the density values in the ROI region were calculated. From the obtained data, Gray-level co-occurrence matrix (GLCM), Neighborhood gray-level different matrix (NGLDM), Gray level run length matrix (GLRLM), and the Gray level zone length matrix (GLZLM) were calculated.We obtained a total of 54 radiomics parameters (**Supplementary Table 3**).

### Statistic Analysis Workflow

First, all the collected samples were randomly divided into test set and validation set according to a ratio of 7:3. We used the sample function of R software to make randomization, and conducted a hypothesis test on the age of the randomized patients between the two groups (**Supplementary Table 4**). The results showed that the average age difference between the two groups was not statistically significant (P > 0.05). Therefore, we selected the group accounting for 70% as the training set for the follow-up analysis of the model. Then, the signatures from image texture were filtered by least absolute and Selection Operator (LASSO) (13). After 100 repeated simulations, signatures with the best robustness were selected. In order to optimize the model, we use the random forest to further screen the selected signatures and obtain the final machine learning model. We performed a multivariate Cox regression analysis of radiological parameters and clinical characteristics and drew a nomogram. The survival curve was plotted by Kaplan-Meier analysis and tested by log-rank test.

## Results

### Establishment of a Model Using Radiomics Signatures

**Figure 2:**
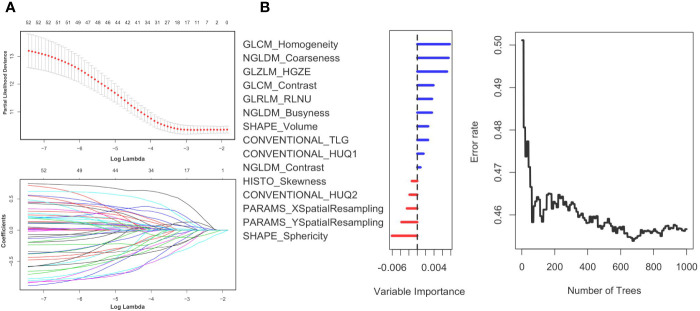
Panel **(A)** shows the Lasso result. Panel **(B)** shows the random forest result. The left **(B)** shows the order of the out-of-bag importance of the selected parameters. The right picture shows relationship between the error rate and the number of classification trees.

**Figure 3:**
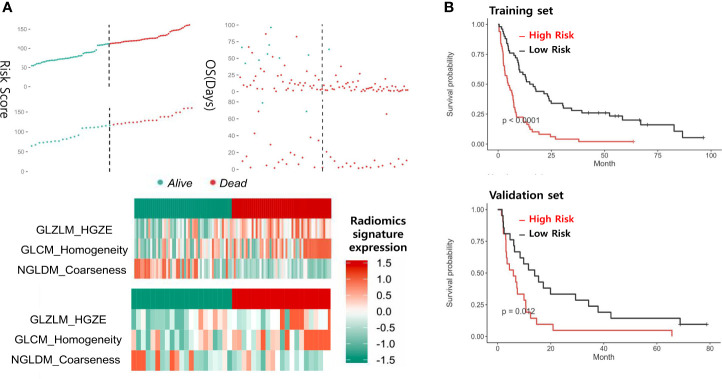
Panel **(A)** shows the distribution of risk scores and the values of the three CT parameters in the training and test groups. Panel **(B)** shows the survival of patients at high or low risk after being grouped by median.

First, we randomly divided the patients into a training group and a test group, with a split ratio of 7:3. Then, LASSO method was used to make simulation in the training group for up to 100 times and 15 signatures were selected. The results are shown in **Figure 2**. Next, the random forest was used to further optimize. According to the relative importance, three most important parameters were screened out (**Supplementary Table 2**). Then, we built a model based on random forest algorithm. The risk score for each patient is calculated and the risk score distribution for each patient is shown in **Figure 3**. By comparing the high with low risk groups, we found that the high risk group had a worse overall survival. And GLZLM-HGZE, and GLCM-homogeneity increased risk, but the increase of NGLDM-coarseness reduces the risk, so we think GLZLM-HGZE and GLCM-homogeneity may be a risk factor of GBC, and NGLDM-coarseness is more likely to be a protective factor. Correlation analysis shows that the correlation degree of these three factors is low (**Supplementary Table 5**). The Univariate COX shows the risk score had a hazard ratio of 1.534 (95% CI: 1.078–2.183). Finally, we validated this model in the verification group (**Supplementary Table 1**). The model had a good performance, with high-risk individuals had poorer survival outcomes than low-risk individuals. In addition, the survival rate of high-risk patients was significantly lower than that of low-risk patients (*P* = 0.012).

### Prognostic Model Performed Based on Clinical Data and Radiomics

**Figure 4:**
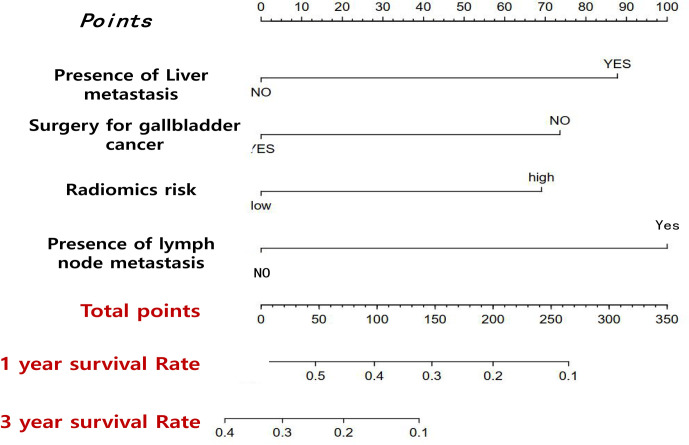
Nomogram that predicts the overall prognosis survival of gallbladder cancer patients after multiple factors are included.

**Figure 5:**
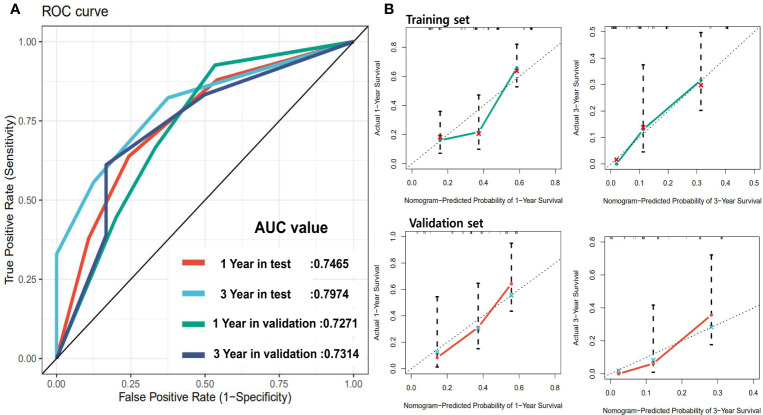
Panel **(A)** shows the ROC of the prognostic survival model incorporating with clinical parameters. Panel **(B)** is the calibration curve of the model.

In order to achieve better performance, we analyzed a variety of clinical indicators of patients, including age, gender, and tumor stage. Through multivariate COX analysis, we screened out the prognostic indicators affecting the survival of patients with GBC, including surgery or not, liver metastasis and lymph node metastasis grade (**Table 2**). Next, we randomly divided the patients into two groups to conduct model training and testing. We used multivariate COX to predict the overall survival of the patients by combining three selected clinical indicators (Liver metastasis, surgery, and lymph node metastasis grade) and radiomics risk score (**Table 3**). Finally, we use a nomogram to visualize the performance of the model (**Figure 4**) and evaluated the prediction accuracy through ROC curve. The results of nomograms showed that the 1- and 3-year prediction reached 0.7465 and 0.7974 in the training group and 0.7271and 0.7314 in the validation group, respectively. **Figure 5** also shows the comparison between the ideal model and the actual nomogram prediction. The calibration chart shows that the actual model is basically consistent with the ideal model, indicating that our model has a high accuracy.

**Table 2:** The results of a multivariate COX analysis.

	P value	HR	Low 95% CI	High 95% CI
Radiomics Risk Score	0.040	1.495	1.019	2.194
Surgery	0.087	0.672	0.426	1.059
Liver metastasis	0.026	1.615	1.060	2.459
N Stage	0.037	1.797	1.035	3.122
Jaundice	0.834	0.953	0.606	1.498
T stage	0.696	1.223	0.447	3.343
Sex	0.456	0.862	0.582	1.275
Age	0.942	1.02	0.602	1.727

**Table 3:** The results of a multivariate analysis combined with clinical examination and radiologic parameters.

	P value	HR	Low 95.0%CI	High 95.0%CI
Liver metastasis	0.009	1.620	1.126	2.322
Surgery	0.077	0.668	0.427	1.044
Radiomics Risk Score	0.042	1.462	1.014	2.107
N Stage	0.042	1.730	1.020	2.935

## Discussion

In this study, CT scan data combined with machine learning methods was used to predict overall survival outcomes in GBC patients. Firstly, we use LASSO to filtered raw data and acquire a robust parameter set. Then we use the random forest to making further optimization. Finally, three parameters including GLZLM-HGZE, GLCM-homogeneity, and NGLDM-coarseness were obtained. The three parameters were correlated with the prognostic risk of the patients, and the prognostic model got a hazard ratio of 1.549. The clinical parameters analysis results showed that lymph node metastasis grade, surgery or not and liver metastasis situation are prognostic factors. In combination with these indicators and CT risk score, we get a model for prognosis of GBC. Moreover, the 3-year AUC value of the predictive model in the random validation group reached 0.797.

It is of great guiding significance for the selection of treatment options and clinical decision support of patients with GBC to find the key prognostic factors related to the survival time and establish an individual and accurate survival prediction model. At present, the prognosis studies of GBC mainly focus on the clinical and pathological examination (6, 14, 15). There are many factors affecting postoperative survival of GBC patients, including TNM stage of tumor, degree of tumor differentiation, liver infiltration, jaundice, and lymph node dissection. The TNM staging is widely used in clinical, but only includes tumor infiltration depth (T stage), lymph node metastasis (N stage), and distant metastases (M stage), with limited survival prediction value. It only for a class of patients, cannot achieve individualized accurate prediction to adjust subsequent treatment. Many studies have focused on the effect of clinical examination on the prognosis of GBC, including inflammatory factors and nutritional indicators (16, 17). However, the original data of those researches almost come from one laboratory examination, and there may be fluctuations in the same individual during different times. Meanwhile, these markers lack specificity, and the mechanism of the correlation between these indicators and tumor outcome is still to be studied. Moreover, clinical data can only reflect the partial biological manifestations of tumor and lack a comprehensive description of entire tumor lesions.

Radiomics is a promising approach to acquire a large amount of intuitive data through the analysis of entire tumor lesion and metastasis. Compared with the molecular features detected by popular omics techniques such as genomics and proteomics, radiomics can better overcome the temporal and spatial specificity of the whole course of cancer. Meanwhile, texture analysis can provide quantitative and semi-quantitative parameters to reflect tumor heterogeneity, which is of great significance in tumor research (18, 19). However, there are few studies of this type conducted in GBC, ascribed to the uniformed data is acquired in clinical experiment. A variety of medical images are applied in clinical decision-making, mainly including CT scan, contrast-enhanced CT, multi-parameter MRI, and PET-CT. CT is the most common and cost-effective data acquisition of original lesions before treatment among them. Although reconstructed MRI sequences and contrast-enhanced CT have advantages in the identification and differentiation of tumors, these detection methods are still not widely used in most areas of China. In terms of applying machine learning, the heterogeneity of tumor tissues is included in multiple texture parameters, thus, the analysis of texture parameters alone cannot fully reflect the overall characteristics of the tumor. Considering this problem, we believe that a complex model integrating different texture signatures is needed to fully identify the total tumor lesion. Also, the random forest is a powerful machine learning method, which has been proved to be able to implement the correct classification work successfully (20, 21). Adopting the concept of integrated learning, random forest has a good accuracy in current machine learning algorithms by combining multiple decision tree models. Moreover, it can process high-dimensional data and be applied in big data effectively. In particular, it can evaluate the importance of each feature in classification. Therefore, in this study, the random forest can better identify parameters and establish classifiers.

However, our study still has three main limitations. Firstly, analyzing CT scan data alone cannot replace other image acquisition methods (such as enhanced CT, mpMRI, reconstructed multiple sequences ADC, and DTI) in real clinical work. Secondly, limited by the size of the sample, there are not enough enhanced CT data and MRI data, thus, this study was not compared with a variety of advanced scanning techniques. Third, our research was limited by its retrospective data. These findings might have better clinical implications, if confirmed in prospective studies. Forth, the patients included in the study were all from a single center, which may result in the lack of sufficient extensibility of the classifier. Considering the differences of medical institutions in obtaining original images and the differences in manual segmentation of lesions, we cannot guarantee that this machine learning classifier performs well on external data sources. But all of the research methods and analysis used in the study come from open-source data packets, which mean that the analysis process needs to be repeated on other data.

## Conclusion

We found associations between established CT imaging parameters and overall survival. Radiomics-based non-invasive technology represented promising ability in predicting the overall survival of gallbladder carcinoma, although more extensive testing are necessary to perfect this technology in real clinical use.

## Data Availability Statement

The original contributions presented in the study are included in the article/**Supplementary Material**. Further inquiries can be directed to the corresponding authors.

## Ethics Statement

The studies involving human participants were reviewed and approved by Ethics Committee of Sichuan University. The patients/participants provided their written informed consent to participate in this study. Written informed consent was obtained from the individual(s) for the publication of any potentially identifiable images or data included in this article.

## Author Contributions

ZL, XM, and JJ are responsible for conceiving and designing the subject. ZL, GZ, XJ and HZ conduct data analysis and article writing. YZ and HZ are responsible for data processing. ZL and GZ make the same contribution to this paper. All authors contributed to the article and approved the submitted version.

## Conflict of Interest

The authors declare that the research was conducted in the absence of any commercial or financial relationships that could be construed as a potential conflict of interest.

## Supplementary Material

Supplementary Figure 1 Shows an example of ROI segmentation.

Supplementary Table 1 Shows radiomics parameters and prognostic risk scores for all patients.

Supplementary Table 2 Shows the importance of the 15 parameters analyzed by LASSO in the random forest.

Supplementary Table 3 Shows the values of all radiomics parameters for all patients.

Supplementary Table 4 Shows the demographic parameters, some laboratory test parameters and median survival time for all patients.

Supplementary Table 5 Shows the correlation between all image parameters in the model.
