# Supplementary figures and images for: Survival Prediction in Gallbladder Cancer Using CT Based Machine Learning
Source: Front Oncol. 2020 Nov 27;10:604288. doi: 10.3389/fonc.2020.604288 (PMC7729190; doi:10.3389/fonc.2020.604288)

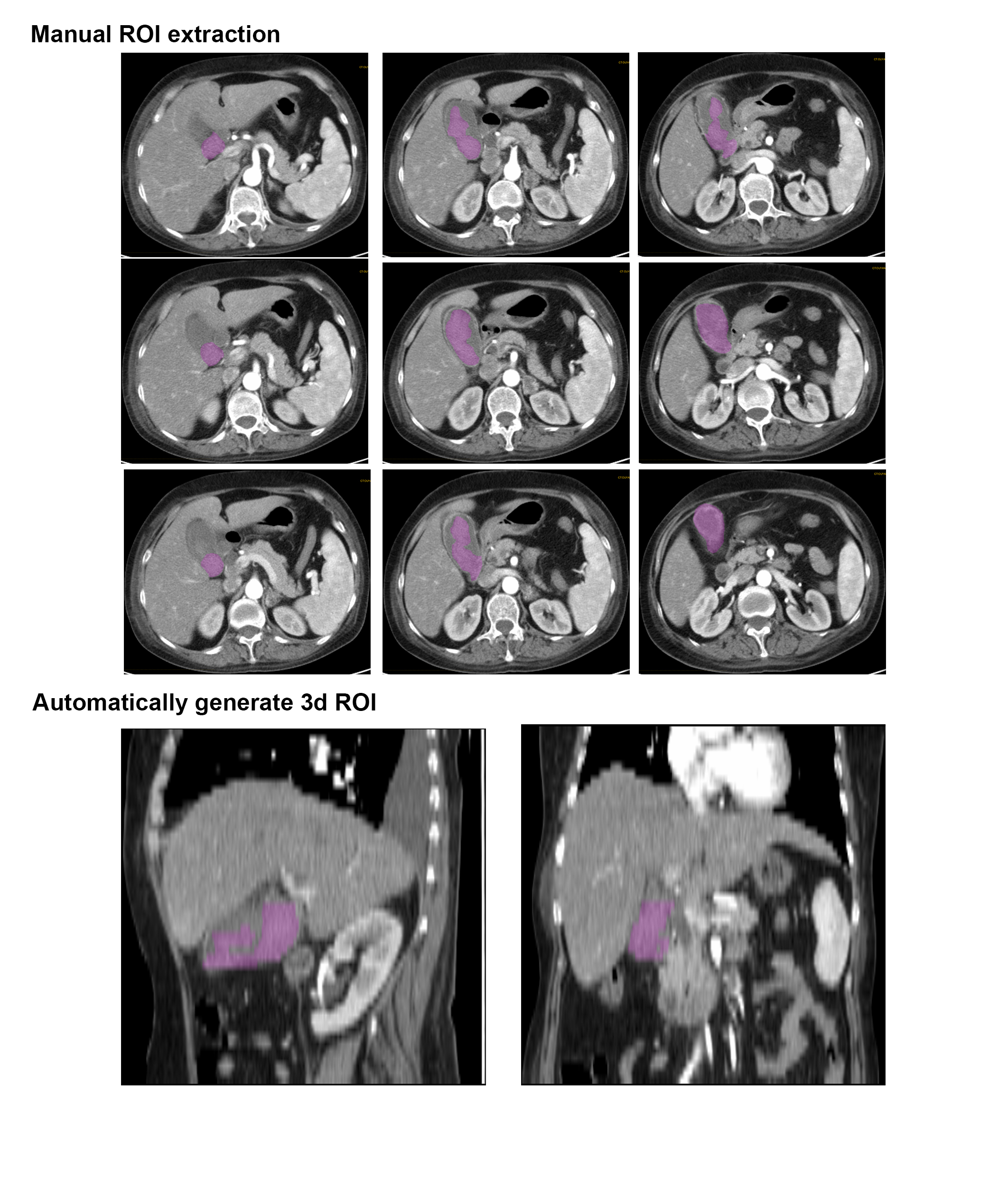

Supplement: Supplementary Figure 1 — Shows an example of ROI segmentation. [file Image_1.jpg]
